# Progression and topographic subtypes of Terrien marginal degeneration

**DOI:** 10.1111/aos.17524

**Published:** 2025-05-19

**Authors:** Minna Ruutila, Pauliina Repo, Annamari T. Immonen, Joni A. Turunen, Marja‐Liisa Lokki, A. Inkeri Lokki, Jukka Moilanen, Kari Krootila, Tero T. Kivelä

**Affiliations:** ^1^ Department of Ophthalmology University of Helsinki and Helsinki University Hospital Helsinki Finland; ^2^ Eye Genetics Group Folkhälsan Research Center Helsinki Finland; ^3^ Transplantation Laboratory, Department of Pathology University of Helsinki Helsinki Finland; ^4^ Department of Bacteriology and Immunology University of Helsinki Helsinki Finland; ^5^ Translational Immunology Research Program, Research Programs Unit University of Helsinki Helsinki Finland; ^6^ Heart and Lung Center Helsinki University Central Hospital Helsinki Finland

**Keywords:** corneal tomography, corneal topography, progression, subtypes, Terrien's marginal degeneration

## Abstract

**Purpose:**

To report long‐term outcomes and to search for immunological and genetic risk factors in Terrien marginal degeneration (TMD).

**Methods:**

Retrospective, in part prospective, hospital‐based longitudinal follow‐up study of 32 eyes of 16 Finnish patients from 2012 to 2023. Median follow‐up was 7.3 years (range, 0.3–15.2). Symptoms, best corrected visual acuity, pattern in axial power map, astigmatism, corneal thickness, higher order irregularity, cavities, progression and human leukocyte antigen genes were analysed. In 13 blood samples, 483 corneal and inflammatory disease‐related genes were analysed with exome sequencing.

**Results:**

The median age at first examination was 61 years (range, 13–89). Eleven (69%) patients were male, and 13 (81%) had bilateral disease. The median annual rate of progression of topographic astigmatism and new thinning was 0.03 D (range, −1.50 to 3.60) and 12.9 μm (range, −107.8 to 93.0), respectively; 0.15 D (range −1.50 to 1.17) and 21.6 μm (range, 1.3–93.0) in 6 (38%) patients with fast progression, and 0.02 D (range, −0.06 to 3.60) and 4.1 μm (range, −107.8 to 24.7) in 10 (72%) patients with slow progression. Topographic pattern, unilaterality, cavities, sectoral hyperaemia, poor response to medical treatment and new thinning after surgery were associated with fast progression. Thickness at maximal thinning fell below 450 μm only with fast progression. Five eyes changed the topographic pattern. Coexisting keratitis fugax hereditaria was found in one patient.

**Conclusions:**

A subtype of TMD progresses faster. The most sensitive indicators of progression were thinning and topographic astigmatism. No shared genetic cause for TMD was identified.

## 1 INTRODUCTION

Terrien's marginal corneal degeneration (TMD) is an infrequent, idiopathic, usually bilateral (56% to 86%), asymmetric and often progressive disease characterised by circumferential peripheral corneal thinning, intact epithelium, neovascularization over the thinned cornea and lipid accumulation anterior to the thinning (Chan et al., 2015; Ruutila et al., 2021; Terrien, 1900; Wang et al., 2015). Patients lack systemic autoimmune diseases, and family members are commonly unaffected, except in two case reports of brothers and cousins (Austin & Brown, 1981; Other, 1966). The literature lacks genetic studies, including human leukocyte antigen genes contributing to immune responses associated with inflammatory eye diseases (Goverdhan et al., 2005).

During our Nordic Terrien Degeneration (NOTED) study proposing clinical diagnostic criteria for TMD, we noticed repeating topography patterns – crab claw, mixed and arcuate – in axial power maps that could change to another pattern during follow‐up as the disease progressed, suggesting different subtypes of the disease (Ruutila et al., 2021, 2023). Knowledge of the long‐term outcome of TMD is limited. Retrospective studies detected progression, inducing 1.7–2.0 D astigmatism or more in 3–5 years, whereas thinning was reported to accelerate 3–5 years after diagnosis (Chan et al., 2015; Das et al., 2021). Seven patients with over 5 years of follow‐up without surgical intervention have been published, of whom five showed progression (Doggart, 1930; Nirankari et al., 1983; Other, 1966; Sanford & Gifford, 1925; Soong et al., 1986). Two publications with over 5 years of follow‐up after lamellar keratoplasty report recurrence (Liang et al., 2008; Pettit, 1991).

Treatment commonly aims to alleviate symptoms, and surgical intervention is typically delayed until imminent perforation. Half of the patients report intermittent redness, mild ocular surface disease symptoms (OSD) and blurring. Conjunctival hyperaemia and blepharitis have also been occasionally reported during examinations (Arnalich‐Montiel, 2024; Chan et al., 2015; Fernandes, 2011; Frank, 1896; Fuchs, 1901; Handmann, 1908; Joseph et al., 1984; Kraupa, 1926; Nirankari et al., 1983; Richards, 1963; Rodriguez et al., 2017; Sanford & Gifford, 1925; Trümpy, 1881). Thus, these patients are often treated with cortisone or cyclosporin drops, which have favourable effects on the redness but without evidence of halting the progression (Arnalich‐Montiel, 2024; Nirankari et al., 1983; Rodriguez et al., 2017). Systemic immunomodulatory treatment has only been reported once for a patient with TMD with no effect on progression but is common in practice in cases of rapid progression (Arnalich‐Montiel, 2024).

A deeper understanding of the course of the disease and its underlying mechanisms is needed to follow up and treat the patients optimally. Thus, the purpose of our present study was to observe the course of the disease in the long‐term and find possible genetic variations associated with it.

## 2 MATERIALS AND METHODS

Our primary aim was to observe patients in the long‐term and find possible genetic risk factors based on retrospectively and, from 2022 onwards, prospectively collected longitudinal data with blood samples from 16 Finnish patients. The secondary objectives were to find the best outcome measures for progression, detect possible prognostic factors and survey treatment response to current treatment protocols. The study was approved by the review board of the Head and Neck Center, Helsinki University Hospital, Finland (HUS/2496/2016). It followed the tenets of the Declaration of Helsinki.

All patients underwent a comprehensive ophthalmic examination including best spectacle corrected visual acuity (BSCVA), slit lamp biomicroscopy, indirect ophthalmoscopy, corneal photography and corneal tomography. We diagnosed TMD retrospectively and prospectively during the NOTED study between 2012 and 2016 and one patient later in 2019. Our clinical diagnostic criteria for TMD were published in collaboration with Swedish and Danish investigators (Ruutila et al., 2021). In brief, we defined TMD by peripheral corneal thinning, lipid deposits at the leading edge, vascularization over the thinned corneal periphery and mild bulbar paralimbal conjunctival hyperaemia without other inflammatory findings. The epithelium had to be intact and the patient free of autoimmune diseases.

Inclusion criteria of the present study additionally included the availability of corneal tomographic follow‐up data recorded with the Anterior Segment OCT Casia SS‐1000 (Tomey, Nagoya, Japan), a 3‐dimensional non‐contact swept‐source optical coherent tomography (AS‐OCT). It provides high‐resolution imaging with 10 μm axial and 30 μm transverse precision and high‐speed scanning with 30 000 A‐scans per second. All scans were taken in 3D mode, which measures 10 mm diameter corneal maps and 16 mm diameter scans by default. Thirty‐two eyes of 16 patients fulfilled the inclusion criteria. We defined fast progression as an increase in the thinned area, new thinning in AS‐OCT at the corneal periphery exceeding 50 μm in less than 4 months, or an increase >1 D in topographic astigmatism.

HLA‐A, HLA‐B and HLA‐DRB1 from the 16 patients were analysed as part of their autoimmune disease screening. HLA genes were determined using a commercial Olerup HLA‐A‐B‐DR SSP Combi Tray typing kit (101.701‐24u/06u – without Taq polymerase, Olerup, Vienna, Austria) with low to intermediate allele resolution. The control population data comprised 150 unselected voluntary Finns completing a health survey before starting a new job, as previously reported (Seppanen et al., 2006).

We invited the enrolled patients for follow‐up examination, unless they were still monitored in the corneal unit and re‐examined all of them between July 2022 and April 2023. Two patients, #15 and #16, had previously passed away, and one, #5, could not be contacted. Thus, 13 patients came for re‐examination, of whom 7 were for study purposes only. They all gave blood samples after written informed consent.

We recorded ocular symptoms and all treatment aimed at TMD. History of other eye surgeries, ocular comorbidities, eye infections or inflammations, systemic diseases, family history of autoimmune diseases and details of autoimmune disease screening are published in the NOTED study with patient numbers listed in Table 1. We tested corneal sensitivity by gently touching the central cornea with a cotton swab. Sensitivity was graded as qualitatively normal (the patient blinked) or slightly reduced (delayed blink reflex, the patient reported sensation). We classified the axial power maps of each eye according to the topographic pattern described in our previous article as crab claw (CC), mixed (M), arcuate (A) and normal by visual grading and consensus of 2 experienced corneal specialists (K.K. and J.M.) masked to patient identifying records (Ruutila et al., 2023). We recorded the presence of peripheral stromal cavities and corneal thickness at the maximal thinning, in addition to regular astigmatism and higher‐order irregularity (HOI) 3 mm from the vertex by Casia SS‐1000. HOI was recorded for the anterior and posterior surfaces in addition to their combined effect (total) at a 3 mm diameter around the vertex. Maximal corneal thinning was often located outside the topographic scans, thus measured by hand in cross‐sectional AS‐OCT scans with the epithelium. The peripheral area with the maximal thinning was first measured from the latest scans and then from the same location as in earlier scans.

**TABLE 1:** General characteristics, symptoms, treatment and corneal sensitivity at the last follow‐up examination of 15 patients with Terrien's marginal degeneration ordered by age at the time of the last examination.

Patient no./sex/age, y/no. in noted study^1^	Laterality	Follow‐up time, years	Symptoms	Inflammatory findings	Progression	Treatment	Corneal sensitivity	Cavities
1/M/28/1	OD	15.2	Mild occasional OSD	None	Yes/Healthy	Corneoscleral rim patch graft, MTX, drops: FML, AT	Normal	Yes
2/F/36/4	OU	10.7	Mild OSD and intermittent redness OU	Intermittent sectoral hyperaemia	Yes/No	MTX, INX, drops: CsA, DEX, PA, FML, RMX, AT	Normal OU	Yes
3/M/45/6	OU	10.3	Mild occasional OSD	Blepharitis	No/No	AT, LH	Normal OU	No
4/M/46/5	OD	10.4	Mild occasional OSD	None	Yes/Healthy	None	Normal OU	No
5/M/50/11	OU	0.3	Mild occasional OSD, previous blurring OU	None	Yes/Yes	CsA drops	Slightly reduced OU	Yes
6/M/64/14	OS	9.2	Mild occasional OSD	One‐off sectoral conjunctival hyperaemia	No/Yes	MTX, drops: FML, AT	Normal	Yes
7/M/65/16	OU	7.5	Mild occasional OSD and blepharitis	Blepharitis and hyperaemia	No/No	AT, LH	Normal OU	No
8/M/68/17	OU	7.2	Visual blurring in the OS	Blepharitis and sectoral hyperaemia	Yes/Yes	MTX, DOX, rim transplant OS, drops: DEX, FML, AT	Normal OU	Yes
9/F/72/18	OU	6.6	Mild occasional OSD	None	No/No	AT	Normal OU	No
10/F/76/21	OU	8.4	Mild occasional OSD	None	No/No	Drops: FML, AT	Normal OU	No
11/F/78/22	OU	6.0	Mild occasional OSD	Blepharitis	No/No	AT	Normal OU	No
12/F/79/−^a^	OU	7.2	Mild occasional OSD, redness and blepharitis	Blepharitis and hyperaemia	No/No	Drops: HCPCS, AT	Slightly reduced OU	No
13/M/81/25	Bilateral	6.4	Mild occasional OSD	None	No/No	AT	Normal OU	No
14/M/81/24	Bilateral	8.4	Mild occasional OSD and blepharitis	Blepharitis and hyperaemia	No/No	AT	Normal OU	No
15/M/82/26	Bilateral	0.3	Mild occasional OSD	None	No/No	AT	Normal OU	No
16/M/90/27	Bilateral	0.7	Mild OSD, redness	None	No/No	AT	Slightly reduced OU	No

Abbreviations: AT, artificial tears; CsA, cyclosporine; DEX, dexamethasone; DOX, doxycycline; F, female; FML, fluorometholone; HCPCS, hydrocortisone sodium phosphate; INX, infliximab; LH, lid hygiene; M, male; OS, oculus sinister; OSD, ocular surface disease; OU, oculus uterque; PA, prednisolone acetate; RMX, rimexolone; y, year.

^a^ Ocular comorbidities: no, family history of autoimmune diseases: no, autoimmune disease screening: normal (ACE, serum angiotensin‐converting enzyme; ANA, antinuclear antibodies; ANCA, anti‐neutrophil cytoplasmic antibodies; CBC, complete blood count; CRP, C‐reactive protein; ESR, erythrocyte sedimentation rate; LZM, lysozyme; RF, rheumatoid factor).

**FIGURE 1:**
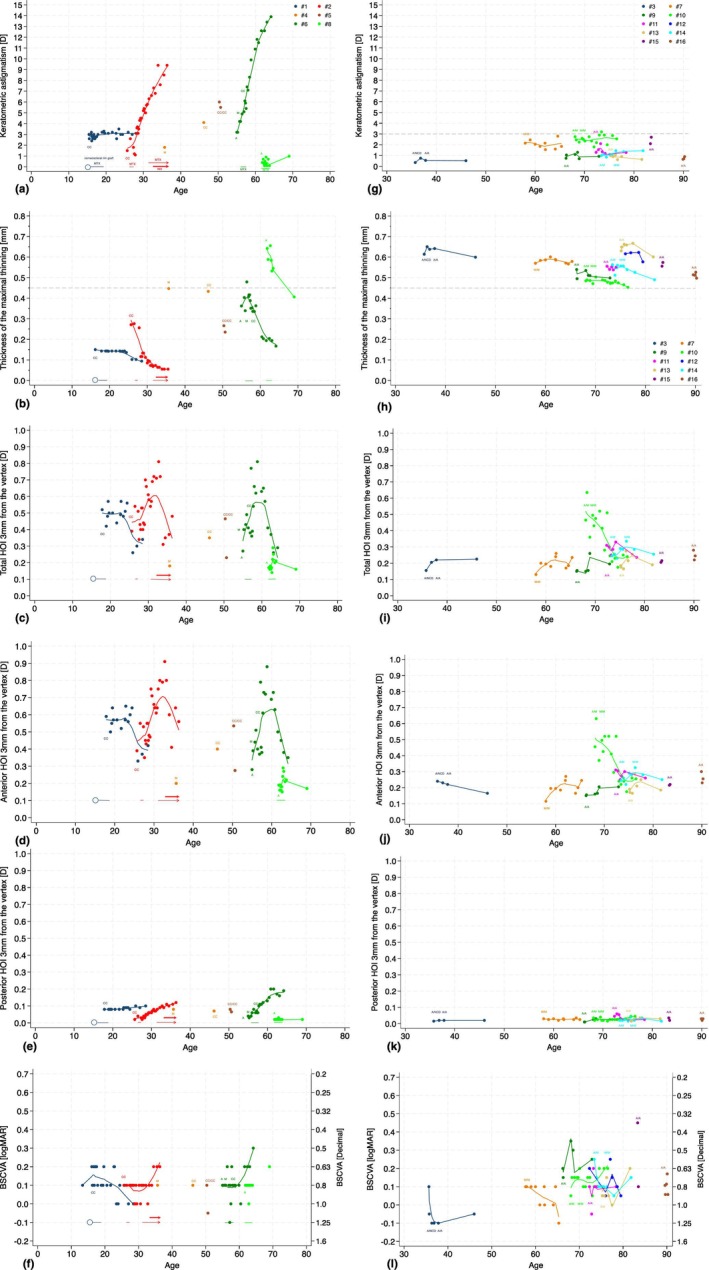
Imaging parameters and best spectacle corrected visual acuity (BSCVA) by age in seven eyes of six patients showing progression, but not perforated (a–f) and both eyes of ten slowly progressing (g–l) patients with Terrien's marginal degeneration. Patterns and their change during follow‐up are marked with CC (crab claw), M (mixed), A (arcuate) and NCD (normal topography in clinical disease). Initiation and discontinuation of methotrexate (MTX, thin horizontal line), infliximab (INX, thick horizontal line) and sclerocorneal rim transplantation (circle) are indicated at the bottom of the graph. An arrow at the end of the horizontal line indicates continuation of medication beyond follow‐up.

The data were analysed graphically by plotting tomographic variables and BSCVA against age (Stata 17, Stata Corp., College Station, TX). Decimal visual acuities were converted to logMAR, and finger counting vision was converted as previously suggested for statistical analyses (Schulze‐Bonsel et al., 2006). Patients suffering from fast progressive disease were analysed separately, taking into account only the progressing non‐perforated eyes. Data of patients having bilateral fast progressive or slowly progressive disease were reported as the mean of both eyes at the time of examination. We applied non‐parametric line fit using locally weighted scatterplot smoothing (lowess) to analyse trends over time (Figure 1). Fisher's exact test was used to compare proportions, and Mann–Whitney *U* test was used to compare continuous variables. A 2‐tailed *p* value < 0.05 was considered significant. False discovery rate (FDR) according to Benjamini‐Hochberg was used to correct for multiple comparisons of allele frequencies (*q*‐value, data not shown). Statistical allele frequency comparison was done in R (R v4.3.3, The R Foundation, Vienna, Austria) using the package exact 2 × 2.

DNA for exome sequencing was isolated from peripheral whole blood samples using a Blood DNA Isolation Kit (Geneaid Biotech, New Taipei City, Taiwan) following the manufacturer's instructions. CeGat (Tübingen, Germany) performed exome sequencing. Sequencing libraries were prepared with the Twist Human Core Exome combined with RefSeq and Mitochondrial Panels (Twist Bioscience, South San Francisco, CA) and sequenced using the NovaSeq 6000 platform (Illumina, San Diego, CA) with 2 × 101 bp reads. Variants were called using the DRAGEN platform (software version 4.2.4, Illumina) to GRCh38. Variant annotation and analysis were done using the Omnomics next‐generation sequencing platform (Euformatics, Espoo, Finland) utilising the Ensembl Variant Effect Predictor version 107.0 (Cambridge, United Kingdom).

A panel of 483 genes (Table S1) associated with corneal abnormalities, corneal dystrophies, autoinflammatory disorders, or immunodeficiency was analysed for variants with minor allele frequency (MAF) ≤ 0.01 in the gnomAD database (v.2.1.1 for exomes and 3.1.2 for genomes) (Karczewski et al., 2020). Genes were selected from the literature (Table S1), and panels for primary immunodeficiency or monogenic inflammatory bowel disease (Version 7.15), autoinflammatory disorders (Version 2.3), corneal abnormalities (Version 1.13) and corneal dystrophy (Version 3.10) are available at https://panelapp.genomicsengland.co.uk by PanelApp. Variants with variant allele frequency < 20%, read depth < 10, and benign or likely benign Clinvar entries were excluded. The American College of Medical Genetics (ACMG) classification for remaining coding region variants (exons ± 20 bp) was obtained using VarSome (Saphetor, Lausanne, Switzerland), and those that received a benign or likely benign classification were excluded from further analysis (Richards et al., 2015). SpliceAI prediction was obtained from https://spliceailookup.broadinstitute.org (Jaganathan et al., 2019). Additionally, all variants classified as pathogenic or likely pathogenic according to ACMG guidelines by Omnomics were inspected for potential association with TMD and further analysed using Varsome and scientific literature. The remaining variants were inspected using Integrative Genomics Viewer (IGV) to exclude sequencing artefacts. An updated gnomAD v.4.1.0 MAF was retrieved for the remaining variants from https://gnomad.broadinstitute.org. The phasing of the *TTC7A* variants was done using samples from the #9 patient's two children who reported no symptoms or findings indicating TMD. Corresponding genomic regions of *TTC7A* were amplified with PCR, followed by sequence analysis using Sanger sequencing. Further details are available upon request.

## 3 RESULTS

All 16 patients were native Finns, and fast progression was detected in 6 patients (38%, 95% CI 18–61; Table 1). Thirteen patients had bilateral disease (81%, 95% CI 54–96); 3 of the 6 patients with fast progressing disease as compared to all 10 patients with slowly progressing disease (*p* = 0.04, Fisher's exact test). Eleven (69%, 95% CI 44–86) were male. The male‐to‐female ratio was 5:1 in patients with fast progressive and 6:4 in slowly progressive disease (*p* = 0.59).

The median age at first examination of all patients was 61 years (range, 13–89), 43 years (range, 13–61) in fast‐progressing patients, and 70 years (range, 35–89) in slowly progressive ones (*p* = 0.008, Mann–Whitney *U* test). The median interval between the first and the last examination was 7.3 years (range, 0.3–15.2); 9.8 years (range, 0.3–15.2) for fast progressing and 6.9 years (range, 0.25–10.3) for slowly progressing patients (*p* = 0.10, Table 1).

### 3.1 Clinical findings

Corneal sensitivity was slightly reduced in three (19%, 95% CI 6–44) patients; in 1 of 6 patients with fast progressing disease, and in 2 of 10 patients with slowly progressing disease (*p* > 0.99, Fisher's exact test). All patients reported symptoms: 15 (94%, 95% CI 70–100) patients reported mild occasional OSD symptoms, lacking in one patient with progressive disease (*p* = 0.38) and 2 reported visual blur, both with fast progressing disease (*p* = 0.13).

Intermittent sectoral hyperaemia, lacking in patients with slow progression, was detected in three patients (19%, 95% CI 6–44) with fast progressing disease (*p* = 0.04, Fisher's exact test). However, one‐off sectoral hyperaemia was nasal and bilateral in patient #6 with unilateral disease; inferior in patient #8 with bilateral thinning mostly in superior quadrants; and temporal in patient #2 with bilateral disease showing unilateral progression during the follow‐up, with most thinning superiorly. Blepharitis was detected in 6 (38%, 95% CI 18–61) patients; in 1 of 6 patients with fast progression, and in 5 of 10 patients with slowly progressing disease (*p* = 0.31). Patients with diffuse hyperaemia had slowly progressing disease and blepharitis. (Table 1).

All six patients with fast‐progressing disease and none of those with slowly progressing disease had the CC pattern in their progressing eyes, except one who had an A pattern and three spontaneous perforations in the fellow eye (*p* = 0.001, Fisher's exact test). However, the pattern of patient #12 with slowly progressive disease was undefinable because of bilateral nasal pterygium. Cavities were present in 7 of the 29 eyes with clinical disease (24%, CI 95% 10–40). Moreover, the six patients with fast‐progressing disease had stromal cavities in their progressing eyes, except #4; whereas patients with slowly progressive disease did not have cavities (*p* = 0.001). The number of eyes with spontaneous perforation was one in a patient with fast progression and zero in patients with slow progression (*p* = 0.38).

### 3.2 Longitudinal findings

Median regular astigmatism in topography at first examination was 2.4 D (range, 0.4–6.0), and 4.8 D (range 0.98–13.9) at last examination in patients with fast progressing disease and 1.1 D (range, 0.35–2.75) and 1.3 D (range 0.5–2.6) in patients with slowly progressing disease, not exceeding 3 D at any point of follow‐up, except in one outlier (*p* = 0.14 at first examination, *p* = 0.008 at last examination, Mann–Whitney *U* test, Figure 1a,g). The median rate of progression of all patients was 0.03 D per year (range, −1.50 to 3.60; IQR 0.74 to −0.02); 0.15 D per year (range −1.50 to 1.17; IQR 0.85 to −0.37) in patients with fast progression, and 0.02 D per year (range, −0.06 to 3.60; IQR 0.40 to −0.03) in those with slow progression (*p* = 0.53).

Median thickness, with epithelium, at the maximal peripheral thinning at first examination was 317 μm (range, 143–669) in patients with fast progression and 560 μm (range, 485–630) in patients with slow progression (*p* = 0.04, Mann–Whitney *U* test), 201 μm (range, 94–433) and 563 μm (range, 453–601), respectively, at last examination (*p* < 0.001). Furthermore, the thickness fell below 450 μm in all patients with fast progression but not in patients with slow progression throughout the follow‐up (Figure 1b,h). The median rate of the thinning of all patients was 12.9 μm per year (range, −107.8 to 93.0; IQR −1.7 to −21.8); 21.6 μm per year (range, 1.3–93; IQR −3.8 to −50.8) in patients with fast progression, and 4.1 μm per year (range, −107.8 to 24.7; IQR −0.8 to −9.3) in patients with slow progression (*p* = 0.093). Slow thinning of the corneal periphery over the years was typical of patients with slowly progressive disease not leading to visual impairment or other complications.

The topographic pattern changed in the two eyes of two patients with fast‐progressing disease from A to M and later to CC in patient #6 and from M to CC in patient #4 (Figure 1a–f). Among patients with slowly progressive disease, one eye changed from normal to A and two eyes of two other patients changed from A to M (Figure 1g–l). In all 5 eyes changing pattern, total higher order irregularity (HOI) increased before taking a downward trend. The HOI of the posterior corneal surface gradually increased in eyes with fast‐progressing disease and remained stable in eyes with slowly progressing disease (Figure 1c–e,i–k). In addition, patients with fast‐progressing disease had more HOI of the anterior and posterior cornea compared to patients with slowly progressing disease.

Analysing trends in fast progressing patients with more than two examinations was possible in 3 eyes of 3 patients (#2, #6 and #8). Increased topographic astigmatism and new thinning were the most sensitive indicators of progression, followed by increasing HOI and change in pattern, if not CC already. Moreover, the peaking of HOI preceded the deterioration of BSCVA in all of these patients.

BSCVA of the eyes with fast‐progressing disease decreased because of rising regular and irregular astigmatism but did not become worse than 20/40. However, subjective refraction varies and is affected by other ocular comorbidities increasing with age, making it an insensitive marker of progression (Figure 1f,l). The eye of patient #8 underwent three non‐symptomatic spontaneous perforations and received one corneoscleral rim transplant, still retaining BSCVA of 20/32.

All 10 patients with slowly progressing disease received artificial tears, two received corticosteroid drops and two received advice for lid hygiene. Four of six patients with fast progressive disease received topical and systemic immune modulatory treatment (IMT), one received cyclosporin drops and two received corneoscleral rim transplants (listed in detail in Table 1 and marked by age in Figure 1). Figure 1a–f shows how the disease progresses in three of the four patients despite topical cortisone, cyclosporine and systemic treatment with methotrexate and infliximab. Moreover, new thinning adjacent to and at the corneoscleral graft of patient #1 was observed in the months following surgery, and progressive thinning continued after corneoscleral transplant in patient #8.

### 3.3 Human leukocyte antigen allele frequencies

Compared to alleles of Finnish controls, HLA‐A*24, HLA‐B*44 and HLA‐DRB1*13 might be relatively more common in slowly progressive disease. The modest sample size did not allow for conclusive analyses, but a trend emerges, where patients with fast progressive disease may more often have the HLA‐A*32, HLA‐B*08 and HLA‐DRB1*01 alleles (Tables S2 and S3, Figure S1).

### 3.4 Exome sequencing

No common genetic aetiology was identified in Finnish patients with TMD, either within the analysed genes associated with corneal or immune‐related disorders (Table S1) or among variants classified as pathogenic or likely pathogenic outside the panel by automated Omnomics analysis (Table S4). The latter variants were scrutinised for potential novel genetic associations with TMD. Two patients, #7 and #9, were found to carry variants linked to inflammatory diseases; and complement factor C2 deletion, detected in patients #1 and #3, is known to associate with age‐related macular degeneration (Kwong et al., 2024).

Patient #7, with slowly progressing TMD, had the heterozygous c.61G>C; p.(Asp21His) variant in *NLRP3*, causing dominantly inherited keratitis fugax hereditaria (KFH, Table S1) (Turunen et al., 2018). Varsome interprets this variant to have unknown significance (VUS) based on its allele frequency in gnomAD, but it is a known disease‐causing variant enriched in Finnish and Swedish populations (Immonen et al., 2022). The patient's medical records do not mention KFH, although presumed anterior uveitis was documented at ages 51 and 58. Laboratory tests for uveitis, including HLA‐B27, were negative. Following the genetic finding, a detailed inquiry into childhood ocular symptoms revealed a history of recurrent red, painful and light‐sensitive eyes since adolescence. No significant vision loss was noted during or after attacks, indicating mild KFH. One of the patient's two children also reported occasional eye symptoms suggestive of KFH. No other TMD patients were identified with the *NLRP3* variant c.61G>C or another likely pathogenic *NLRP3* variant.

Patient #7 was identified with a variant c.1879G>T p.(Glu627*) of unknown significance (VUS) in *IFIH1*, causing dominant interferonopathy (Table S1), not matching the patient's clinical history.

Patient #9 was found to be compound heterozygous for two recessive *TTC7A* missense variants, c.1817A>G; p.(Lys606Arg) and c.2014T>C; p.(Ser672Pro). Varsome classifies both variants as benign due to their high allele frequency in the general population and computational predictions. However, the variants have been reported to co‐occur in *trans* in a patient with a mild phenotype of intestinal atresia and combined immunodeficiency, and in *cis* with a loss‐of‐function allele in a patient with a severe phenotype (Chen et al., 2013; Lawless et al., 2017). Sequencing revealed that neither variant was identified in the descendants, confirming they are in *cis* and patient #9 is not at risk for the recessive condition associated with these alleles. Additionally, the patient was heterozygous for a likely pathogenic *DLX3* variant c.481del p.(Glu161Serfs*13) (Table S4). *DLX3* was not included in the panel of genes of interest as pathogenic variants in the gene cause dominantly inherited trichodento‐osseous syndrome (Liu et al., 2022). No evidence of a *DLX3*‐associated phenotype was noted in the medical records.

Additional pathogenic or likely pathogenic variants in recessive genes within the panel, as well as variants of unknown significance in dominant genes, are listed in Table S1, while pathogenic or likely pathogenic recessive variants outside the gene panel are reported in Table S4.

## 4 DISCUSSION

To the best of our knowledge, our study is the longest follow‐up study in the literature and the first exome‐based genetic analysis of TMD. It is the first part of a prospective follow‐up study, which likely reduces sources of bias and confounding factors. We defined fast progression as an accelerated peripheral thinning or >1 D increase in topographic astigmatism. Risk indicators for fast‐progressing disease were younger age at diagnosis, unilaterality, cavities, CC topographic pattern, increasing topographic astigmatism, thinning of the corneal periphery below 450 μm and sectoral hyperaemia. Patients with fast‐progressing disease were treated more actively but had poor responses to topical and systemic IMT, in addition to recurrence after surgery, consistent with a case study on systemic mycophenolate mofetil and three case series reporting recurrence after lamellar keratoplasty (Arnalich‐Montiel, 2024; Li et al., 2018; Liang et al., 2008; Pettit, 1991). Age under 60 years, male predominance, visual blur and high‐grade HOI were other repeated findings. In contrast, patients with slowly progressing disease were commonly over 60 years old, had less than 3 D of topographic astigmatism, thickness at the maximal thinning exceeding 450 μm, stable HOI of the posterior cornea and less than 1 D change in topographic astigmatism despite pattern change. BSCVA was an insensitive marker of progression, especially in the elderly with other ocular comorbidities.

Comparison of the present series with the three largest ones reporting the rate of progression is shown in Table S5 (Chan et al., 2015; Das et al., 2021; Ruutila et al., 2021). A cross‐sectional retrospective hospital‐based study of 285 eyes of 184 patients from India reported topographic follow‐up of 170 eyes, some with follow‐up of up to 5 years after diagnosis (Das et al., 2021). However, it remains unclear whether it is the series with the longest follow‐up because of missing data about the number of eyes and patients in each group with different follow‐up times. Our study was comparable in sex, laterality and eyes with perforations, pseudopterygiums and corneal grafts. Main differences compared to our series were the absence of peripheral corneal thinning in over half of the patients, corneal scarring, young age, lack of systemic autoimmune disease screening and a low number of eyes with blepharitis and dry eye disease. Our NOTED study of 29 Nordic patients, including 15 patients from the present study, is the second largest series with potentially the longest follow‐up, median 3 years (range, 0–18) (Ruutila et al., 2021). The present series were comparable in age, sex, laterality, presence of thinning in all eyes, absence of scarring, cavities, perforations, eyes requiring corneal grafts and eyes with pseudopterygiums. NOTED reported irritation‐related symptoms, hyperaemia and blepharitis to be less frequent compared to the present Finnish series. The difference is likely due to the different study designs, retrospective vs. prospective, the latter being more reliable. Moreover, the patients were older than in NOTED by the end of the present follow‐up, increasing the probability of OSD and blepharitis.

Furthermore, the present series was comparable with the third largest and the third longest follow‐up series of 25 patients and a median of 2.5 years of follow‐up from Canada in age, sex, laterality, presence of thinning, absence of scarring, eyes with perforations, hyperaemia, blepharitis, eyes with pseudopterygiums and eyes requiring corneal grafts (Chan et al., 2015). The main differences with our series were the lack of systemic autoimmune disease screening and cavities being less frequent in the Canadian series. The median follow‐up was longer, and the progression of topographic astigmatism per year was slower in our series compared to all previous ones.

In our previous report regarding topographic patterns, higher order aberrations (HOA) increased with age and the posterior cornea showed more HOA in the CC pattern, as a group, compared to the other patterns. In addition, increasing HOAs were associated with a reduction in BSCVA in the CC pattern as a group (Ruutila et al., 2023). In the present series, we detected increasing HOI of the posterior cornea throughout follow‐up, a reduction of BSCVA followed by peaking of total HOI with a delay and a high grade of total HOI, first increasing before taking a downward slope in patients with fast progressive disease and the CC topographic pattern.

We previously supposed that patterns in axial power maps reflect the stage of the disease (Ruutila et al., 2023). Change of pattern in 5 eyes of 5 patients during the present study from normal to A, M and CC was detected, not favouring either group by progression rate, except for the CC pattern found in patients with fast progression. The change in pattern was associated with accelerated peripheral thinning, increasing topographic astigmatism and increasing HOI in all 5 eyes, being more evident in eyes with fast progression. However, the non‐perforated eye of patient #8 progressed and had cavities without changing from the pattern A. Thus, while a change in pattern indicates progression, it fails to show progression in some patients and in patients already with the CC pattern. Thus, it is insufficient as the only outcome measure. It is possible, even likely, that we have missed A and M patterns that potentially preceded CC in patients with late diagnosis who may have had more aggressive disease when they were younger.

Based on clinical findings, two types of TMD have been suggested: inflammatory and quiescent (Austin & Brown, 1981; Iwamoto et al., 1972; Lauber, 1905; Nirankari et al., 1983; Rodriguez et al., 2017). Many of these studies lack autoimmune disease screening or do not fulfil the clinical diagnostic criteria as later defined in our NOTED study. All our patients, except for one with fast progression, reported OSD symptoms, which seem to be an insensitive marker of progressive disease. Furthermore, blepharitis was as common as in the Canadian series and was not associated with fast progression (Chan et al., 2015). Sectoral hyperaemia, detected occasionally in half of our patients with progressive disease, was commonly located elsewhere than adjacent to the meridian with maximal thinning, which speaks against sclerokeratitis, which also commonly would be accompanied by pain and swelling. Scleritis has been reported to co‐exist with TMD in publications that did not exclude systemic autoimmune diseases (Austin & Brown, 1981; François, 1936). Exceptionally, in the Canadian series, scleral thinning without bulbar inflammation was detected in one patient requiring surgery (Chan et al., 2015).

Lamellar keratoplasty (LK) and patch grafts have become the mainstay in the surgical treatment of TMD (Ding et al., 2019). However, studies monitoring BSCVA and astigmatism with over 2 years of follow‐up postoperatively are few and report recurrence (Hattori et al., 2013; Li et al., 2018; Liang et al., 2008; Pettit, 1991). Our patients #1 and #8 showed progression after the corneoscleral patch graft. Thus, surgery should not be considered a curative treatment. Spontaneous perforations of patient #8 were asymptomatic despite normal central corneal sensitivity, consistent with the findings of Terrien that the peripheral changes are insensitive (Terrien, 1900).

The aetiology of TMD is unknown, but the most common hypotheses are degeneration and inflammation. Paralimbal location of the thinning suggests immunologic origin because the peripheral cornea is closer to the immunologically active conjunctiva (Mondino, 1988). However, patients with TMD lack specific recurring inflammatory findings by histology, electron microscopy, immunohistochemistry, IVCM, angiography of peripheral vessels and serum immunocomplex studies (Berkowitz et al., 1983; Guyer et al., 1987; Lopez et al., 1991; Pouliquen, Dhermy, et al., 1989; Pouliquen, Renard, & Savoldelli, 1989; Rodriguez et al., 2017; Ruutila et al., 2024; Skribek et al., 2008; Süveges et al., 1972; Watson & Bovey, 1985).

Although familial occurrences of TMD have been reported on two occasions (Austin & Brown, 1981; Other, 1966), sequence analysis of variants in genes associated with corneal and immune‐related disorders in our 13 Finnish patients with TMD revealed no shared monogenic germline cause. Patient #7 harboured the *NLRP3* variant c.61G>C, causing KFH (Turunen et al., 2018), an autoinflammatory disease characterised by recurrent attacks during which the corneal stroma becomes infiltrated with leukocytes with some spillover to the anterior chamber. The attacks typically begin during childhood and reoccur a few times annually (Immonen et al., 2022; Ruusuvaara & Setala, 1987; Valle, 1964, 1966). KFH is often misdiagnosed as mild anterior uveitis, which was also the case in the medical records of the variant carrier (Immonen et al., 2022; Kawan et al., 2024; Turunen et al., 2020). The KFH manifested as an unusually mild form with no visual blur and no permanent corneal opacities. About half of the patients with KFH develop central stromal opacities, but this patient is the only one with TMD (Immonen et al., 2022). Our finding is likely coincidental, reflecting the high frequency of KFH in Finland and we do not suspect an association between KFH and TMD (Immonen et al., 2022; Kivela, 2022).

To the best of our knowledge, our study is the first to describe the long‐term course of the subtypes of fast‐ and slow‐progressing TMD (Ruutila et al., 2023). Our study has limitations because of its partially retrospective nature, small sample size, subjectivity in visual grading of the patterns and limited follow‐up time. Also, the patients had variable ocular comorbidities that were often the reason for their original referral. Patients with fast and slowly progressing disease were analysed differently, taking into account the progressing but non‐perforated eyes in the first group and the mean of both eyes in the latter group, potentially making the progression of patients with fast progressive disease more evident, whereas potentially having a smoothing effect in patients with slowly progressing disease. The disease was commonly unilateral and more asymmetric in patients with fast progression compared to patients with slow progression, all of whom had bilateral and more symmetric disease.

Bilateral uneventful LASIK was performed in patient #4 11 years before unilateral TMD was diagnosed, which may have altered corneal biomechanics; however, no flap displacement or other complications were detected. Cataract surgery was performed in 3 eyes of 2 patients, #13 and #16, before TMD was diagnosed and in 4 eyes of 2 patients, #9 and #15, after the diagnosis. However, these corneas had the A pattern, which has not been described to be associated with past cataract surgery and neither patient showed a change in pattern because of surgery. Additionally, our genetic analysis has limitations: variants located in genes outside the panel, within introns, or hard‐to‐sequence regions, as well as variants not flagged as deleterious or undetectable by short‐read sequencing, such as large genomic rearrangements, may have gone undetected. HLA genes located on chromosome 6 are the most polymorphic within the human genome and are known to be associated with eye disease. Selecting Finns as the control population regarding HLA alleles reduced the bias related to population‐based variation in allele frequencies (Wennerstrom et al., 2013). The genotyping results, particularly in the fast‐progressing group, are inconclusive due to the small number of samples available. A larger number of patients will be needed to decipher if HLA‐A*24 or other alleles are associated with TMD subtypes and in non‐Finnish patients.

In conclusion, the identification of the disease type enables us to study new efficient treatments while curbing the need to expose patients to ineffective or even harmful ones.

## FUNDING INFORMATION

This study was supported by grants from the Finnish Eye Foundation, the Eye and Tissue Bank Foundation, the Mary and Georg C. Ehrnrooth Foundation, and the Evald and Hilda Nissi Foundation.

The funder had no role in the design and conduct of the study; collection, management, analysis and interpretation of the data; preparation, review, or approval of the manuscript; and decision to submit the manuscript for publication.

The authors wish to acknowledge the key contribution of late Prof. Juha Holopainen, MD, FEBO, Department of Ophthalmology, University of Helsinki and Helsinki University Hospital, the initiator of the NOTED study, who passed away before preparation of the manuscript.

## CONFLICT OF INTEREST STATEMENT

Minna Ruutila, Kari Krootila and Tero T. Kivelä report personal fees from Santen Finland outside the submitted work. Joni Turunen reported personal fees unrelated to the current work from Santen Finland, Thea Finland and Macular Finland. For the remaining authors, none is declared.

## Supporting information


Figure S1.

Table S1.

Table S2.

Table S3.

Table S4.

Table S5.
