# Supplementary figures and images for: Progression and topographic subtypes of Terrien marginal degeneration
Source: Acta Ophthalmol. 2025 May 19;104(1):33–43. doi: 10.1111/aos.17524 (PMC12803575; doi:10.1111/aos.17524)

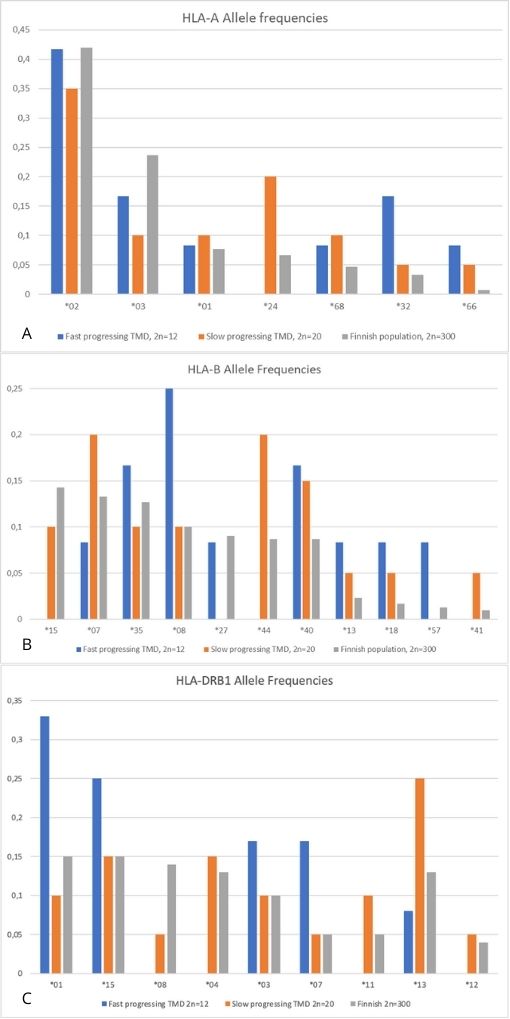

Supplement: Supplementary file 1 — Figure S1. [file AOS-104-33-s004.jpg]
