# Supplementary material for: Progression and topographic subtypes of Terrien marginal degeneration
Source: Acta Ophthalmol. 2025 May 19;104(1):33–43. doi: 10.1111/aos.17524 (PMC12803575; doi:10.1111/aos.17524)
Supplement: Supplementary file 2 — Table S1. [file AOS-104-33-s002.docx]

**Figure** **S1.** Graphical comparison of HLA-A (A), HLA-B (B) and HLA-DRB1 (C) alleles of slow and fast progressing TMD to the Finnish control population sample. Alleles that are observed in >5% of the Finnish population (*n* = 150) or observed in TMD patients are included.

| **Table S1.** Variants in genes associated with corneal dystrophies, corneal abnormalities or autoinflammatory syndromes. All were identified as heterozygous and were filtered to include those with gnomAD allele frequency ≤0.01. | | | | | | | | | | |
| --- | --- | --- | --- | --- | --- | --- | --- | --- | --- | --- |
| **Germline variant (transcript)** | **Chromosomal position**  **(Hg38)** | **SpliceAi** | **ACMG classification**  **(applied rules)** | **gnomAD v.4.1.0 MAF** | **Gene associated condition (source)** | **ClinVar variation** | | | **Patient** | **Phenotype** |
|  |  |  |  |  |  | **ID** | **Interpretation (count)** | |  |  |
| *C2* (ENST00000299367.10) c.841_849+19del | chr6-31934289-TG…TC (28bp)- | Δ score Donor Loss 0.21 | LP  (PVS1, PP5, PS3, BS2) | ALL 0.0057 FIN 0.0054 | AR Complement Component 2 Deficiency (PubMed: 1577763) | 50634 | | Pathogenic (13)  Likely pathogenic (3)  Uncertain significance (1) | 1 | Fast progressive TMD |
| *STS* (ENST00000674429.1)  c.340G>A p.(Ala114Thr) | chrX-7257546-G-A | Δ scores <0.2 | VUS (PM2) | absent | XLR Ichthyosis (CCID:007950) | n/a | | | 2 | Fast progressive TMD |
| *C2* (ENST00000299367.10) c.841_849+19del | chr6-31934289-TG…TC (28bp)- | Δ score Donor Loss 0.21 | LP  (PVS1, PP5, PS3, BS2) | ALL 0.0057 FIN 0.0054 | AR Complement Component 2 Deficiency (PubMed: 1577763) | 50634 | Pathogenic (13)  Likely pathogenic (3) Uncertain significance (1) | | 3 | Slowly progressive TMD |
| *OAS1* (ENST00000202917.10)  c.531C>A p.(Cys177*) | chr12-112911112-C-A | Δ scores <0.2 | VUS  (PVS1, BS2) | ALL 0.000027 FIN 0.00055 | AD autoinflammatory immunodeficiency  (PMID: 34145065) | n/a | | | 4 | Fast progressive TMD |
| *PEPD* (ENST00000244137.12) c.1135G>A p.(Val379Met) | chr19-33391312-C-T | Δ scores <0.2 | VUS  (PM2. PP3. BP1) | ALL 0.000020 FIN 0.00033 | AR prolidase deficiency  (CCID:007640) | 1401107 | Uncertain significance (1) | | 6 | Fast progressive TMD |
| *GINS1* (ENST00000262460.5)  c.247C>T p.(Arg83Cys) | chr20-25418112-C-T | Δ scores <0.2 | VUS  (PP5, PS3, PM2, BP4) | ALL 0.00066 FIN 0.0040 | AR growth retardation and combined immune deficiency  (PubMed:28414293, PubMed: 31630891) | 487511 | Pathogenic (3)  Likely pathogenic (3)  Uncertain significance (1) | | 7 | Slowly progressive TMD,  mild KFH, osteoarthritis |
| *IFIH1* (ENST00000649979.2)  c.1879G>T p.(Glu627*) | chr2-162277580-C-A | Δ scores <0.2 | VUS  (PVS1, PP5, BS2, BP6) | ALL 0.0052 FIN 0.0021 | AD IFIH1-related type 1 interferonopathy  (CCID:008354) | 377048 | Uncertain significance(1)  Likely benign (5)  Benign (2) | |  |  |
| *NLRP3* (NM_004895.5)  c.61G>C p.(Asp21His) | chr1-247418855-G-C | Δ scores <0.2 | VUS  (BS2, PP5, PP2, PS3) | ALL 0.0000081 FIN 0.00014 | AD Keratitis fugax hereditaria  (PubMed:29366613) AD Cryopyrin associated periodic syndrome (PMID: 31077002) | 495298 | Likely pathogenic (1)  Pathogenic (1) | |  |  |
| *LIG1* (ENST00000263274.12)  c.776+5G>T | chr19-48149758-C-A | Δ score Donor Loss 0.97 (+5bp) Δ score Donor Gain 0.52 (-11bp) | VUS  (PP3, PM2, BP6) | ALL 0.00037 FIN 0.0059 | AR Severe Combined Immune Deficiency (PubMed:30395541) | 1145405 | Likely Benign (1) | | 8 | Fast progressive TMD |
| *LOXHD1* (ENST00000642948.1)  c.197C>G p.(Thr66Arg) | chr18-46649203-G-C | Δ scores <0.2 | VUS  (BP4, PM1, PM2) | ALL 0.00053 FIN 0.00087 | AD Late-Onset Fuchs Corneal Dystrophy (PMID: 22341973)  AR Nonsyndromic hearing loss  (ClinGen CCID:005297) | 286763 | | Uncertain significance (5) | 9 | Slowly progressive TMD, hypertension |
| *TTC7A* (ENST00000319190.11)  c.1817A>G p.(Lys606Arg)  c.2014T>C p.(Ser672Pro) | chr2-47046329-A-G | Δ scores <0.2 | B  (BS1, BS2, BP1, BP4, PP5) | ALL 0.0030 FIN 0.0011 | AR multiple intestinal atresia and combined immunodeficiency (CCID:006464) | 242606 | | Uncertain significance(5) Likely benign (3) |  |  |
|  | chr2-47050043-T-C | Δ scores <0.2 | B  (BS2, BP1, BP4, PP5) | ALL 0.0030 FIN 0.0011 |  | 242605 | | Uncertain significance(5)  Likely benign(3) |  |  |
| *NHEJ1* (ENST00000356853.10)  c.653A>C p.(Tyr218Ser) | chr2-219078142-T-G | Δ scores <0.2 | VUS  (PP3, PM2) | ALL 0.000023 FIN 0.00055 | AR Cernunnos-XLF deficiency (CCID:005640) | n/a | | | 10 | Slowly progressive TMD, hypertension |
| *CD3D* (ENST00000300692.9)  c.465del p.(Asp156Metfs*24) | chr11-118339213-T- | Δ score <0.2 | LP  (PVS1, PM2) | ALL 0.000037 FIN 0.00062 | AR immunodeficiency 19 (CCID:004375) | n/a | | | 11 | Slowly progressive TMD |
| *HPS1* (ENST00000361490.9)  c.972del p.(Met325Trpfs*6) | chr10-98427230-G- | Δ score <0.2 | P  (PVS1, PP5, PM2) | ALL 0.000053 FIN 0.00056 | AR Hermansky-Pudlak syndrome (CCID:005083) | 5280 | Pathogenic (9)  Not provided (1) | | 13 | Slowly progressive TMD |
| *STAT3* (ENST00000264657.10)  c.670C>T p.(Leu224Phe) | chr17-42337562-G-A | Δ scores <0.2 | VUS  (PM2, PP3) | absent | AD hyper-IgE recurrent infection syndrome 1 (CCID:006284) AD STAT3-related early-onset multisystem autoimmune disease (CCID:006283) | n/a | | | 14 | Slowly progressive TMD,  Hypertrophic cardiomyopathy, hypertension |
| AD = autosomal dominant; AR = autosomal recessive; B = benign; BP1 = missense variant in a gene for which primarily truncating variants are known to cause disease; BP4 = multiple lines of computational evidence suggest no impact on gene or gene product (conservation, evolutionary, splicing impact, etc.); BP6 = reputable source recently reports variant as benign, but the evidence is not available to the laboratory to perform an independent evaluation; BS1 = allele frequency is greater than expected for disorder; BS2 = variant observed in a healthy adult individual for a recessive (homozygous), dominant (heterozygous), or X-linked (hemizygous) disorder, with full penetrance expected at an early age; CCID = ClinGen curation identification; FIN = Finnish population; KFH= Keratitis fugax hereditaria; LP = likely pathogenic; MAF = minor allele frequency; n/a = not available; P = pathogenic; PM1 = located in a mutational hot spot and/or critical and well-established functional domain (e.g., active site of an enzyme) without benign variation; PM2 = variant is absent from controls (or at extremely low frequency if recessive) in Exome Sequencing Project, 1000 Genomes Project, or Exome Aggregation Consortium; PP2 = missense variant in a gene that has a low rate of benign missense variation and in which missense variants are a common mechanism of disease; PP5 = reputable source recently reports variant as pathogenic, but the evidence is not available to the laboratory to perform an independent evaluation; PS3 = well-established in vitro or in vivo functional studies supportive of a damaging effect on the gene or gene product; PVS1 = null variant (nonsense, frameshift, canonical ±1 or 2 splice sites, initiation codon, single or multiexon deletion) in a gene where LOF is a known mechanism of disease; TMD = Terrien’s marginal degeneration; VUS = variant of unknown significance; XLR = X-linked recessive | | | | | | | | | | |
