# Supplementary material for: Progression and topographic subtypes of Terrien marginal degeneration
Source: Acta Ophthalmol. 2025 May 19;104(1):33–43. doi: 10.1111/aos.17524 (PMC12803575; doi:10.1111/aos.17524)
Supplement: Supplementary file 3 — Table S2. [file AOS-104-33-s001.docx]

**Table S2**. Systemic diseases, immunological and demographic data of fast progressive and slowly progressive patients by age with Terrien’s marginal degeneration

| Progression | ID | Sex | HLA-A | HLA-B | HLA-DRB1 | Systemic diseases |
| --- | --- | --- | --- | --- | --- | --- |
| Fast | #1 | M | *02,*66 | *18,*27 | *01,*15 | None |
|  | #2 | F | *03,*03 | *07,*35 | *01,*15 | Migraine |
|  | #4 | M | *02,*02 | *13,*35 | *01,*07 | None |
|  | #5 | M | *02,*32 | *08,*40 | *01,*03 | None |
|  | #6 | M | *01,*32 | *08,*40 | *13,*15 | None |
|  | #8 | M | *02,*68 | *08,*57 | *03,*07 | HA, DM2 |
| Slow | #3 | M | *25,*68 | *07,*18 | *15,*15 | None |
|  | #7 | M | *02,*24 | *44,*44 | *04,*12 | OA |
|  | #9 | F | *01,*02 | *07,*08 | *03,*11 | HA |
|  | #10 | F | *02,*24 | *07,*35 | *13,*15 | HA |
|  | #11 | F | *66,*68 | *35,*41 | *01,*13 | None |
|  | #12 | F | *02,*32 | *15,*44 | *04,*04 | HA |
|  | #13 | M | *03,*24 | *07,*40 | *13,*13 | HA, DM2, CAD |
|  | #14 | M | *02,*03 | *13,*15 | *07,*08 | HCM, AF, HA, CVA |
|  | #15 | M | *02,*24 | *40,*40 | *01,*13 | DM2, gout, HCL, CVA |
|  | #16 | M | *01,*02 | *08,*44 | *03,*11 | HA, AF, COPD |

Abbreviations: CAD, coronary artery disease; CVA, cerebrovascular accident; COPD, chronic obstructive pulmonary disease; DM2, diabetes mellitus type 2; F, female; AF, atrial fibrillation; HA, hypertensio arterialis; HCL, hypercholesterolemia; HCM, Hypertrophic cardiomyopathy; HLA, human leucocyte antigen; M, male; NA, not available; OA, osteoarthritis
