# Supplementary material for: Progression and topographic subtypes of Terrien marginal degeneration
Source: Acta Ophthalmol. 2025 May 19;104(1):33–43. doi: 10.1111/aos.17524 (PMC12803575; doi:10.1111/aos.17524)
Supplement: Supplementary file 4 — Table S3. [file AOS-104-33-s003.docx]

**Table S3.** Statistical comparison of slow and fast progressing TMD to Finnish population sample. Alleles that are observed in >5% of Finnish population (N=150) or observed in TMD patients are included. Allele frequences for Finnish population are from Wennerström et al. 2013

|  | Frequency | | | p-value | |  |
| --- | --- | --- | --- | --- | --- | --- |
| HLA-A allele | Fast progression 2n=12 | Slow progression 2n=20 | Finnish 2n=300 | Fast progression vs Finnish | Slow progression vs Finnish |  |
| **02* | 0.42 | 0.35 | 0.42 | 1 | 0.643 |  |
| **03* | 0.17 | 0.10 | 0.24 | 0.739 | 0.268 |  |
| **01* | 0.08 | 0.10 | 0.08 | 1 | 0.662 |  |
| **24* | 0 | 0.20 | 0.07 | 1 | 0.052 |  |
| **68* | 0.08 | 0.10 | 0.05 | 0.452 | 0.263 |  |
| **32* | 0.17 | 0.05 | 0.03 | 0.072 | 0.514 |  |
| **66* | 0.08 | 0.05 | 0.01 | 0.111 | 0.177 |  |
| **25* | 0 | 0.05 | <0.01 | 1 | 0.121 |  |
| HLA-B allele | Fast progression 2n=12 | Slow progression 2n=20 | Finnish 2n=300 | Fast progression vs Finnish | Slow progression vs Finnish |  |
| **15* | 0 | 0.10 | 0.14 | 0.383 | 1 |  |
| **07* | 0.08 | 0.20 | 0.13 | 0.497 | 1 |  |
| **35* | 0.17 | 0.10 | 0.13 | 0.657 | 1 |  |
| **08* | 0.25 | 0.10 | 0.10 | 0.122 | 1 |  |
| **27* | 0.08 | 0 | 0.09 | 1 | 0.394 |  |
| **44* | 0 | 0.20 | 0.09 | 0.609 | 0.105 |  |
| **40* | 0.17 | 0.15 | 0.09 | 0.294 | 0.408 |  |
| **13* | 0.08 | 0.05 | 0.02 | 0.272 | 0.407 |  |
| **18* | 0.08 | 0.05 | 0.02 | 0.211 | 0.323 |  |
| **57* | 0.08 | 0 | 0.01 | 0.179 | 1 |  |
| **41* | 0 | 0.05 | 0.01 | 1 | 0.229 |  |
| HLA-DRB1 allele | | Fast progression 2n=12 | Slow progression 2n=20 | Finnish 2n=300 | Fast progression vs Finnish | Slow progression vs Finnish |
| **01* | | 0.33 | 0.1 | 0.15 | 0.101 | 1 |
| **15* | | 0.25 | 0.15 | 0.15 | 0.400 | 1 |
| **08* | | 0 | 0.05 | 0.14 | 0.378 | 0.491 |
| **04* | | 0 | 0.15 | 0.13 | 0.376 | 0.740 |
| **03* | | 0.17 | 0.1 | 0.10 | 0.339 | 1 |
| **07* | | 0.17 | 0.05 | 0.05 | 0.147 | 1 |
| **11* | | 0 | 0.1 | 0.05 | 1 | 0.288 |
| **13* | | 0.08 | 0.25 | 0.13 | 0.429 | 0.176 |
| **12* | | 0 | 0.05 | 0.04 | 1 | 0.546 |
