# Supplementary material for: Progression and topographic subtypes of Terrien marginal degeneration
Source: Acta Ophthalmol. 2025 May 19;104(1):33–43. doi: 10.1111/aos.17524 (PMC12803575; doi:10.1111/aos.17524)
Supplement: Supplementary file 5 — Table S4. [file AOS-104-33-s005.docx]

| Table S4. Variants classified as pathogenic (P) or likely pathogenic (LP) by Omnomics automated ACMG classification. Variants that received benign or likely benign classification with Varsome were excluded. All variants were identified as heterozygous. | | | | | | | | | |
| --- | --- | --- | --- | --- | --- | --- | --- | --- | --- |
| **Germline variant (transcript)** | **Chromosomal position (Hg38)** | **SpliceAi** | **ACMG classification**  **(applied rules)** | **gnomAD v.4.1.0 MAF** | **Gene associated condition (source)** | **ClinVar** | | **Patient** | **Phenotype** |
|  |  |  |  |  |  | **ID** | **Interpretation (count)** |  |  |
| *FAM217B* (ENST00000360816.8)  c.602del p.(Ala201Glufs*18) | chr20-59944545-C- | Δ score <0.2 | VUS (PM2) | ALL 0.000028 FIN 0.00059 | n/a | n/a | | 4 | Fast progressive TMD |
| *STRC*  (ENST00000450892.7)  c.4027C>T p.(Gln1343*) | chr15-43604750-G-A | Δ score Acceptor Loss 0.41 Δ score Donor loss 0.42 | P  (PVS1, PP5, PM2) | ALL 0.000091 FIN 0.00017 | AR nonsyndromic hearing loss (CCID:006290) | 448908 | Pathogenic (3)  Likely Pathogenic (2) |  |  |
| *ERCC5* (ENST00000652225.2)  c.2299C>T p.(Gln767*) | chr13-102866361-C-T | Δ score <0.2 | LP  (PVS1, PM2) | ALL 0.00000062 FIN 0.000016 | AR xeroderma pigmentosum group G (CCID:004764) | n/a | | 7 | Slowly progressive TMD |
| *IQCE* (ENST00000402050.7)  c.1614_1617del p.(Lys539Leufs*24) | chr7-2601446-AAAG- | Δ score <0.2 | VUS  (PVS1, PM2) | ALL 0.000011 FIN 0.000016 | AR Polydactyly (PMID: 31549751) | n/a | | 8 | Fast progressive TMD |
| *DLX3* (ENST00000434704.2)  c.481del p.(Glu161Serfs*13) | chr17-49993435-C- | Δ score <0.2 | LP  (PVS1, PM2) | absent | AD Trichodentoosseous Syndrome (PubMed:35714441) | n/a | | 9 | Slowly progressive TMD, hypertension |
| *CPLANE1* (ENST00000651892.2)  c.7915C>T p.(Gln2639*) | chr5-37157766-G-A | Δ score <0.2 | LP  (PVS1, PM2) | ALL 0.0000043 FIN 0.000078 | AR Joubert syndrome 17 (CCID:004566) | n/a | | 10 | Slowly progressive TMD, hypertension |
| *POLR1B* (ENST00000263331.10)  c.1136del p.(Gln379Argfs*7) | chr2-112552794-A- | Δ score <0.2 | LP  (PVS1, PM2) | ALL 0.0000031 FIN 0.000078 | AD Treacher Collins syndrome type 4 (PubMed:31649276) | n/a | |  |  |
| *CEP290* (ENST00000552810.6)  c.1933A>T p.(Lys645*) | chr12-88114539-T-A | Δ score Acceptor Loss 0.22 | LP  (PVS1, PM2) | ALL 0.0000013 FIN 0.000034 | AR CEP290-related ciliopathy (CCID:004417) | n/a | | 11 | Slowly progressive TMD |
| *KRTAP19-3* (ENST00000334063.6)  c.161del p.(Gly54Aspfs*38) | chr21-30491797-C- | Δ score <0.2 | VUS  (PM2) | ALL 0.0015 FIN 0.0043 | n/a | n/a | |  |  |
| NEB (ENST00000397345.8)  c.24209_24212dup p.(Leu8071Phefs*30) | chr2-151497714--AACA | Δ score Acceptor Loss 0.21 | P  (PVS1, PP5, PM2) | ALL 0.000015 FIN 0.000032 | AR Nemaline myopathy (CCID:005608) | 465579 | Pathogenic (5)  Likely pathogenic (2) | 12 | Slowly progressive TMD |
| *PKD1L1* (ENST00000289672.7)  c.7687-2A>G | chr7-47808389-T-C | Δ score Acceptor Loss 0.92  Δ score Donor loss 0.67 | P  (PVS1, PP5, PM2) | ALL 0.000027 FIN 0.00031 | AR Visceral heterotaxy (PubMed:27616478) | 2636016 | Likely pathogenic (1) |  |  |
| *ALMS1* (ENST00000613296.6)  c.8161C>T p.(Arg2721*) | chr2-73490120-C-T | Δ score <0.2 | P  (PVS1, PP5, PM2) | ALL 0.0000087 FIN absent | AR Alström Syndrome (CCID:004109) | 3975 | Pathogenic (4)  Uncertain Significance (1) | 14 | Slowly progressive TMD,  Hypertrophic cardiomyopathy, hypertension |
| AD = autosomal dominant; AR = autosomal recessive; CCID = ClinGen curation identification; FIN = Finnish population; LP = likely pathogenic; MAF = minor allele frequency; n/a = not available; P = pathogenic; PM2 = variant is absent from controls (or at extremely low frequency if recessive) in Exome Sequencing Project, 1000 Genomes Project, or Exome Aggregation Consortium; PP5 = reputable source recently reports variant as pathogenic, but the evidence is not available to the laboratory to perform an independent evaluation; PVS1 = null variant (nonsense, frameshift, canonical ±1 or 2 splice sites, initiation codon, single or multiexon deletion) in a gene where LOF is a known mechanism of disease, TMD = Terrien’s marginal degeneration; VUS = variant of unknown significance; | | | | | | | | | |
