# Supplementary material for: Progression and topographic subtypes of Terrien marginal degeneration
Source: Acta Ophthalmol. 2025 May 19;104(1):33–43. doi: 10.1111/aos.17524 (PMC12803575; doi:10.1111/aos.17524)
Supplement: Supplementary file 6 — Table S5. [file AOS-104-33-s006.docx]

**Table S5.** **Comparison of the present series with the previous follow-up series reporting the rate of progression in Terrien’s marginal degeneration**

| **Variable** | **Indian study by Das et al.** | **p-value** | **Canadian study by Chan et al.** | **p-value** | **Nordic study by Ruutila et al.** | **p-value** |
| --- | --- | --- | --- | --- | --- | --- |
| **N** | 184 patients, 285 eyes |  | 25 patients, 43 eyes |  | 29 patients, 49 eyes |  |
| **Median follow-up time** | NA (range 0 to over 5 years) |  | 30.3 months |  | 3 years |  |
| **Age** |  | *0.004 |  | *0.85 |  | *0.08 |
| **Autoimmune disease screening** | No |  | No |  | Yes |  |
| **Sex** |  | °0.61 |  | °0.52 |  | °0.75 |
| **Laterality** |  | °0.06 |  | °0.71 |  | °0.49 |
| **Thinning** |  | °<0.001 |  | >0.99 |  | °>0.99 |
| **Scaring** |  | °<0.001 |  | >0.99 |  | °>0.99 |
| **Cavities** |  | NA |  | °0.03 |  | °>0.99 |
| **perforations** |  | °0.25 |  | °0.39 |  | °>0.99 |
| **OSD symptoms** |  | °<0.001 | NA | NA |  | °<0.001 |
| **Hyperemia** |  | NA |  | °0.34 |  | °0.05 |
| **Blepharitis** |  | °<0.001 |  | °>0.99 |  | °0.001 |
| **Eyes requiring corneal grafts** |  | °0.31 |  | °0.11 |  | °>0.99 |
| **Pterygiums**  **/pseudopterygiums** |  | °0.08 |  | °>0.99 |  | °0.36 |
| **Progression rate of topographic astigmatism per year** | 0.21D to 0.98 D |  | 0.56 D to 0.75 D |  | 0.41 D |  |

*Mann-Whitney U test

° Fisher’s exact test

Abbreviations: NA, not available; OSD, ocular surface disease
